# Patients’ and researchers’ experiences with a patient board for a clinical trial on urinary tract infections

**DOI:** 10.1186/s40900-019-0172-0

**Published:** 2019-11-28

**Authors:** Imke Schilling, Heike Behrens, Jutta Bleidorn, Ildikó Gágyor, Claudia Hugenschmidt, Hannah Jilani, Guido Schmiemann, Ansgar Gerhardus

**Affiliations:** 1 Department for Health Services Research, Institute of Public Health and Nursing Research, University of Bremen, Grazer Straße 4, 28359 Bremen, Germany; 2 Health Sciences Bremen, University of Bremen, 28359 Bremen, Germany; 3 Institute of General Practice, Jena University Hospital, Bachstr. 18, 07743 Jena, Germany; 4 Institute of General Practice, Medical School Hannover, Carl-Neuberg-Str. 1, 30635 Hannover, Germany; 5 Institute of General Practice, Würzburg University Hospital, Joseph-Schneider-Str.2/D7, 97080 Würzburg, Germany; 6 Institute of General Practice, Göttingen University Hospital, Humboldtallee 38, 37070 Göttingen, Germany

**Keywords:** Clinical trial, Patient and public involvement, PPI, Patient engagement, Patient board, Experiences, Challenges, Qualitative research

## Abstract

**Background:**

Patient and public involvement (PPI) has become an essential part of the design, conduct, and dissemination of research. While researchers who employed PPI mainly report on the positive aspects, in practice PPI is still an exception in clinical trials in Germany. There are specific challenges in the process of involvement that can jeopardize the conduct of involvement. The aim of our study was to analyze the experience of patients and researchers with PPI in a clinical trial in Germany, so we could learn more about potential challenges and how they could be addressed.

**Methods:**

We established a patient board for a randomized controlled trial on urinary tract infections, where patients and researchers regularly met to discuss relevant aspects of the trial. Minutes were taken for each meeting and the moderator also noted her observations in a postscript. After four meetings, we conducted two focus groups, one each with the patients and researchers. We analyzed and categorized the minutes, postscripts, and focus group transcripts using thematic qualitative text analysis.

**Results:**

Patients and researchers felt comfortable with the composition of the patient board and its’ atmosphere. In terms of challenges, patients and researchers needed time to get familiar with PPI. Both parties saw a need for training in PPI but differed in their views on the relevant topics. Patients wished to learn more about their role and tasks within the board at the onset of the PPI. They also preferred to meet more frequently and get more intensely involved in the trial. In contrast, researchers perceived that they were already highly involved. They further felt that the involvement was of benefit to them, the trial and future research. Patients described benefits for themselves, but also wondered if their involvement had had an impact on the trial.

**Conclusions:**

To facilitate effective PPI, resources, adequate structures, and training are needed. Patients and researchers need to agree on their respective roles, training needs, and the mode of cooperation right at the beginning. The parties involved should continuously reflect on the actual benefits of PPI, describe them explicitly and make them transparent for all.

## Plain English summary

Involving patients in the design, conduct and dissemination of research enhances the quality of research and empowers patients. However, there are specific challenges in the process of involvement that can jeopardize the success. To find out about patients’ and researchers’ experiences and challenges with involvement, we set up a patient board for a trial on urinary tract infections. We then analyzed the minutes and postscripts of four patient board meetings, and also conducted two focus groups on patients’ and researchers’ experiences. Patients and researchers felt comfortable with the composition of the patient board and liked the atmosphere during the meetings. In terms of challenges, patients as well as researchers mentioned that they needed time to get familiar with the concept of patient involvement and that training for the involvement was needed. Patients wished to learn more about their roles and tasks within the board at the onset of the involvement. Regarding the timeframe, patients preferred to meet more often so they could become more intensely involved in the trial. In contrast, researchers felt already highly involved. Researchers found that the involvement was to the benefit of researchers, the trial and future research. Patients described benefits for themselves, but they wondered, if their involvement had had an impact on the trial. To conclude, effective patient involvement needs resources, adequate structures, and training. Patients and researchers should agree on their roles and the mode of cooperation at the beginning of the involvement and the benefits of the involvement should be constantly communicated to all parties involved.

## Background

Involving patients and the public in the design, conduct and dissemination of research should become an essential part of research. Patient and public involvement (PPI) is argued to democratize research, empower patients [1–3], and increase the quality and relevance of research [4]. It helps research to focus on the needs of patients, facilitates recruitment, enhances the quality of results, and supports their dissemination [1–3, 5]. Through this, patients benefit from new information, which serves to boost their self-confidence and leads to higher levels of satisfaction [6]. Researchers change their thinking through learning more about patients’ perspectives [7].

While studies on PPI mainly report about its’ positive aspects [1, 3, 7–9], active involvement is still considered to be challenging. This has mainly been accorded to several factors such as differing expectations between researchers and patients [10–12], researchers’ concerns that patients might not be sufficiently equipped to contribute meaningfully, patients’ fears that the involvement is merely tokenistic [3], researchers’ lack of experience with PPI, and the perceived additional efforts associated with the involvement of patients as active partners in the research process [13]. Morain and Forsythe et al. assume that the challenges faced when trying to involve patients and the public in research may be underreported as researchers may fear that reporting these might undermine their chances of getting future funding [14, 15].

In Germany, researchers are increasingly being asked by funding organizations to involve patients in their clinical trials [16, 17]. However, in contrast to other countries, there are no guidelines or frameworks for PPI in the country. In addition, so far little is known about how patients and researchers in Germany experience PPI. Building on our previous work in which we investigated patients’ and researchers’ motivating factors and expectations of getting involved with a patient board [12], we therefore aimed to elucidate the experiences of patients and researchers who were members of a patient board that was established for a clinical trial on urinary tract infections (UTI). By doing so, we wanted to learn about the challenges faced when conducting effective PPI, and discussed patients’ and researchers’ experiences with the patient board, taking their views on their initial motivation and expectations into consideration.

## Methods

For continuity, we use the established, overarching term ‘patient and public involvement’ (PPI) within the text, but are aware that we involved only patients who had experienced UTIs, and not the wider public. We use the term ‘patients’ throughout the article when referring to the people involved in research through contributing their experiential knowledge, as it seems to be the most explicit and easily understood term. We are aware that other terms, e.g. service users, may also fit.

### Establishing the patient board

The study on patients’ and researchers’ experiences with patient involvement was conducted within the context of an ongoing randomized controlled trial (RCT), in which a herbal treatment is compared to a standard (antibiotic) treatment for UTIs in women (EudraCT No. 2016–000477-21) [18]. We established a patient board to support the trial from the preparation phase until the dissemination of the results. In the patient board, patients and researchers regularly met to exchange views, and to discuss relevant aspects of the trial.

### Selection of patient board members

The selection of the patient board members is described in detail elsewhere [12]. In short, ten women (‘patients’), who had experienced UTIs, were included. They were purposefully selected to cover diverse perspectives regarding age, educational background, and experiences with clinical research [5, 19–21]. In addition to the patients, five members of the UTI trial team (‘researchers’) were also involved in the patient board. Informed consent was obtained from both patients and researchers. Each member of the patient board filled out a short questionnaire with socio-demographic data and information on their experience with UTIs (patients) and PPI (patients and researchers).

### General set-up of the patient board

The patient board met regularly every five to seven months. Next to the patients, one researcher of the trial team participated in all meetings of the patient board. The other researchers were invited to join the board meetings as they wished as they lived in a different city (approximately 200 km away). Independent of the patient board, the researchers also held regular trial team meetings every month or two (in person or via telephone).

The form of cooperation within the patient board was discussed and agreed upon during the first patient board meeting. Both patients and researchers could have defined the agenda setting, but in fact, only the researchers and the moderator added issues to the agenda. Issues discussed were for example, the relevance of the research question, patients’ experiences with UTIs and their resultant research interests, the usability of information material for test persons, the planned recruitment strategy and patients’ ideas for a wider recruitment, the dissemination of results and how relevant findings can reach the patients. In most cases the researchers or the moderator prepared a short introduction for the discussion and then invited the patients to share their perspectives and discuss the issues at hand. As the outcomes of the discussions served as the basis for new topics to be discussed in further exchanges, the patients were able to influence the agenda indirectly. The results of the patient board meetings were shared via written minutes and could then be incorporated into the trial by the research team. Patients were not involved in the final decision-making. Applying the continuum of involvement practices as suggested by Forsythe et al. [22], our PPI would qualify as consultation (which would be different from “input” and “collaboration or shared leadership”).

The meetings took place at a venue centrally located in the town of Bremen and were moderated by a researcher (IS), who was not a member of the trial team. Each meeting lasted 120–150 min, including a 15 min break. For every patient board meeting they participated in, patients received an allowance of 50 Euros to cover time and travel expenses. Researchers did not receive any allowances.

### Collecting the experiences of the patient board

The moderator audio-recorded the meetings and the feedback rounds after the meetings and the minutes were transcribed from the audiotapes. Great care was taken that all information that might identify any of the patients was deleted. The resulting documents were counter-checked for accuracy by the participants of the meeting. The final documents were then shared with all patient board members, irrespective of whether they had attended the meeting or not. After each patient board meeting, IS noted her impressions of the meeting in a postscript. The impressions comprised observations regarding the organization of the meeting and the procedure, the atmosphere, the participants, the moderation, the issues discussed, and the feedback received [23].

After four patient board meetings, we conducted two focus groups to discuss the experiences made with PPI, one each with the patients and the researchers. We developed two discussion guides based on the findings of the literature on PPI experiences, our preliminary research on motivations and expectations for PPI, and on the input from the members of the patient board. Both discussion guides addressed the same questions (see Table 1) but had different wording that was customized to the common parlance of each group. The guides were discussed and agreed upon among the authors of this manuscript (six researchers and two patients).

**Table 1:** Questions addressed in the focus groups

Questions addressed in the focus groups	
• Which experiences did you gain on the patient board?	
• What went well, what was not so good?	
• How did you perceive the composition of the patient board?	
• How did you experience the collaboration of patients and researchers?	
• How much effort was involved with the PPI?	
• How did you perceive the impact of the patient board on the trial?	

The focus groups were conducted by the first author (IS), who is female. Each focus group discussion was audio-recorded. The focus group with patients lasted 130 min, and that with researchers 136 min. As for the patient board meetings, the patients received an allowance of 50 Euros for participating and researchers did not receive any allowance.

### Analysis

The data of the focus group discussions were transcribed and pseudonymized by three (student) assistants and checked for accuracy by IS. All written documents from the meetings and focus groups were imported into MAXQDA (Version 11, VERBI GmbH, Berlin, Germany) for analysis. Data analysis of the focus groups was conducted following Kuckartz’ seven steps of thematic qualitative text analysis [24]. We started our analysis by thoroughly reading the transcripts of the focus groups repeatedly, and supplementing them with memos on our research interests, relevant paragraphs and lines of argumentation (step 1).). Primary categories for both patients’ and researchers’ experiences were developed based on the transcripts and memos (step 2). All transcripts were coded according to these categories (step 3). Using the code memos and all text passages of each category (step 4), the preliminary categories were elaborated into final categories and subcategories (step 5). The entire material was then coded again (step 6). The analysis for each category was conducted separately for patients and for researchers (step 7). The analysis was conducted by two of the authors (IS and HJ). IS conducted the seven steps described above and HJ checked the accuracy of coding and category development. Critical aspects were discussed among all authors until an agreement for each aspect was reached. For validation, the findings were sent to all participants, together with a feedback questionnaire asking them whether their perspectives had been adequately described [25, 26]. All feedback questionnaires were returned anonymously and analyzed descriptively. Corrections and amendments were considered in the analysis. The minutes and postscripts were analyzed by IS using the category system developed for the focus groups. The results of the analysis (focus groups, minutes and postscripts) were discussed with the patient and researcher co-authors. The discussion section was drafted by all authors together. We used our previous findings on patients’ and researchers’ motivating factors and expectations of getting involved with the patient board [12] to inform our discussion section. We compared the initial motivation and expectations with the actual experiences of the participants. As the focus group transcripts were in German, citations used in this article were translated into English by IS and back translated by colleagues who were not familiar with the original citations to check the accuracy of translations.

Ethical approval was obtained from the ethics committee of the University of Bremen (Germany). The author team consisted of academic researchers with experience in conducting qualitative research (IS, AG, HJ), women with UTI experiences (CH, HB), and researchers of the RCT (JB, IG and GS) who were involved in the patient board. The reporting was done in accordance with the consolidated criteria for reporting qualitative research (COREQ) (see Additional file 1) [27], and the GRIPP2 checklist for the reporting of PPI (see Additional file 2) [28].

## Results

During the course of the study, two patients left the patient board for personal reasons, and one researcher moved to another project. All three persons were not available for the qualitative study and were therefore not included in the analysis.

The analysis was based on the minutes of four patient board meetings, four reflection postscripts and two focus groups. Seven of the eight remaining patients and three of the four remaining researchers participated in the focus groups. All board members, apart from one researcher, were female. The majority was above 34 years old and had higher education. Only three of the patients and one of the researchers attended all four patient board meetings (Table 2).

**Table 2:** Characteristics of patients and researchers involved in the focus groups

	Patients (*N* = 7)	Researchers (*N* = 3)
Gender		
Female	7	2
Male	0	1
Age		
20–34	2	0
35–49	2	3
50–64	3	0
Educational level		
Higher education	5	3
Secondary education	2	0
Attendance of patient board meetings		
4x	3	1
3x	1	0
2x	3	0
1x	0	2

### Overarching themes and categories

**Fig. 1:**
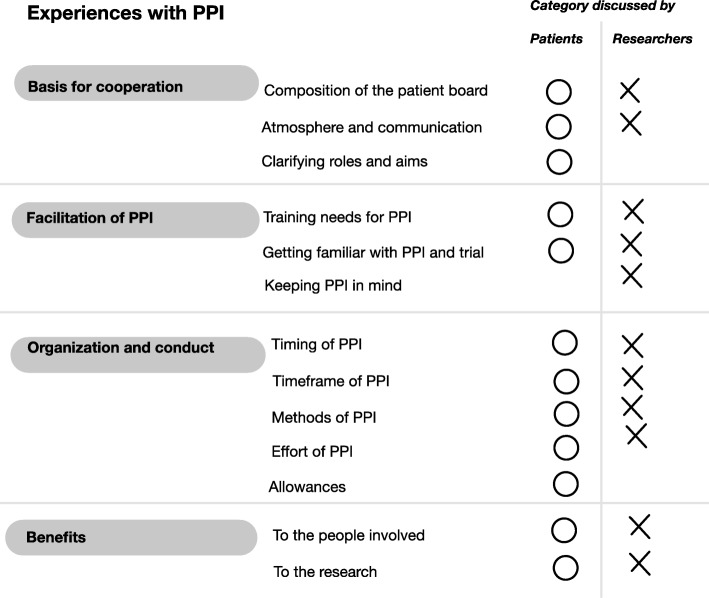
Coding tree

We found four overarching themes that shaped patients’ and researchers’ experiences: “Basis for cooperation”, “facilitation of PPI”, “organization and conduct” and “benefits”. The themes could be further structured into thirteen categories. The topics of ten of these categories were discussed by both groups, and two of the remaining three categories by the patients and the third one by the researchers (see Fig. 1)

### Theme 1: basis for cooperation

Patients and researchers discussed issues that relate to the formation of their cooperation. These comprised the composition of the patient board, the atmosphere and the way of communication within the board, as well as the need to clarify roles and aims for cooperation.

#### Composition of the patient board

Both patients and researchers found the composition of the patient board adequate. Patients appreciated the attendance of the researchers, and that the researchers informed them about the trial and new ideas for the therapy of UTIs. They found it from time to time useful, “[ …] that a physician [researcher] was present and explained some things” (P2:25). Researchers liked the fact that the patients were very diverse in terms of age, professional background, experience with UTI, preferences, and their views on the topics of discussion. When explicitly asked who could be additionally involved in future trials, patients found it reasonable to involve some “women, who actually take part in the clinical trial [ …]” (P3:35). Researchers mentioned involving people who are not affected by UTIs to make the group more heterogeneous.

#### Atmosphere and communication

Patients found the atmosphere on the patient board to be very pleasant, “I had the impression that it was absolutely about involving our perspectives, and that these were respected. I always felt appreciated and taken seriously” (P4:10). They generally felt that the researchers were eager to be at eye level with them, and that they “[ …] could always discuss openly about everything [ …]” (P7:14). Researchers also described the atmosphere in the patient board as being pleasant. They found the “cooperation very constructive and [ …] appreciative” (R3:150). Both patients and researchers appreciated having the moderation. It was reported that the moderator structured the meetings and kept care of all practical needs (snacks and drinks, preparation and follow-up, minutes via audio-recording, scheduling via Doodle, booking of a suitable and central location).

Researchers greatly appreciated having the option to send documents to the board, respectively the moderator, instead of attending the meetings in person. They were also thankful for the timely feedback on the material or any questions they had. Sharing the results of patient board meetings via minutes was experienced as “a good method [ …], that is practical, that is a manageable effort” (R1:171 and 173).

#### Roles and aims

At the beginning of the patient board, all patients wondered about their roles within the board. For some patients, the understanding of their roles and the connected aims developed gradually, over time. Others were still “in search of an answer” (P4:201), even after four meetings. Patients wished to learn more about the terms of reference of the patient board and their supposed role at the beginning of their work. For future patient boards, patients suggested that a) contact persons give more information about the role of the patients when they approach patients for involvement, b) patients receive written information, and c) that patients’ and researchers’ roles be explicitly addressed in the patient board meetings.

Patients drew comparisons between their role on the patient board and the roles of members in self-help groups, and said the difference was that, “[ …] one does not participate [in a patient board] for ones own topics [ …]” (P3:173). Nonetheless, they emphasized that PPI needs to address the own experience to enhance the group formation. Researchers on the other hand did not discuss the issue of roles and aims.

### Theme 2: facilitation of PPI

Three issues that relate to the facilitation of PPI emerged from the patients’ and researchers’ experiences: the training needs for PPI, the need to get familiar with PPI and the trial, and researchers’ difficulties to keep PPI in mind in the context of the clinical trials.

#### Training needs for PPI

Regarding training needs, patients reported that the knowledge they need would depend on the aspects of the trial concerned. For instance, when involved in just checking the comprehensibility of trial materials for patients, they would not need a deeper understanding of the trial; but, “if one shall co-decide upon the research question, one may need a bit more knowledge” (P5:270). The patients did not discuss researchers’ training needs.

The researchers’ views on the PPI training that patients need varied. One researcher stated that “patients need a certain understanding of processes and [ …] the way knowledge is formed” (R1:89), while another preferred to have “real feedback [ …]” , meaning that “[ …] people should say what they think without having a special training” (R3:306). The latter researcher found it more important for the researchers to be well trained in terms of communication, “I think we are required to always look, ‘am I able to get the content across, the question or whatever is important, so that it is understood straightaway’” (R3:306).

#### Getting familiar with PPI and the trial

Patients generally needed time to get familiar with PPI and the trial, and struggled to contribute their own issues during the patient board discussions. According to some of them, being “[ …] involved from the beginning” (P6:262) and “experiencing all steps of the process [ …]” (P1:250) would make it easier to get involved more effectively in the trial. Researchers thought a lot of encouragement and a good moderator with high communication skills were needed to enable patients to share the topics they cared about. As one of them mentioned, “everything [ …] was totally new for everyone [ …] in this patient board. The patients were permanently confronted with new issues, and they were already very much preoccupied with our topics” (R3:325).

While the researchers were already familiar with the trial, they were not familiar with PPI. At the beginning they had no clear understanding of PPI and wondered what it would be like. The experiences they gained through the patient board helped them to fill the term PPI: “[ …] it is definitely more, and better, and richer what I feel now when I work with this term” (R3:273).

#### Keeping PPI in mind in context of the study

The researchers found it challenging to think of the patient board at the right moments; “there is always a standardized process after which we proceed when we plan such a trial [ …] and the patient board was not yet incorporated in it properly” (R3:176). The patients did not discuss this issue.

### Theme 3: organization and conduct

Patients and researchers described their experiences with the organization and conduct of PPI with regard to the timing, the timeframe, the methods, the effort and the allowances.

#### Timing of PPI in context of the study

Both patients and researchers regretted the fact that the patient board only started after the design stage, after the trial had been approved for funding. Patients especially enjoyed one patient board meeting during which the idea for a future UTI trial was discussed from scratch, as the topic was new for everyone and not already completely planned through by the researchers. Similarly, researchers would have preferred to consult with patients “during critical times” (R2:5), when decisions on outcomes and other important aspects had to be taken. Involving patients from the start, “would have influenced us [researchers] more” (R3:9).

#### Timeframe for PPI

The timeframe of the meetings, which ranged from 2 to 2,5 h, was described as the maximum possible, especially if the meetings took place after hours on a working day. For the patients it was important to have enough time to eat after work before attending a patient board meeting. During the meetings a short break helped to maintain concentration.

The interval of five to 7 months between two patient board meetings was found to be too long by the patients as they “forgot the names [of the other patients and researchers] and what had happened in the last [meeting]” (P3:8). They preferred shorter meetings at a higher frequency. Researchers on the other hand thought that holding quarterly meetings might be to ensure continuous work, but stated that the tasks and needs of a clinical trial might not need this many meetings.

#### Methods of PPI

Patients felt that there was not enough direct exchange with the researchers, and described the method of their involvement as, “there was something developed [by the researchers], that was forwarded to us, and we discussed about it” (P6:51). They would have liked to be involved in the whole research process. To enable a more frequent and intense exchange, they suggested that Skype® meetings with the researchers could be organized, or that one to two patients could attend the planning meetings of the trial team and report back to the patient board. Further, the patients preferred personal exchange (also via telephone or Skype®) over written queries; “the interesting aspect is the discussion in the group” (P2:158). They felt that the discussions were more fruitful when they focused on practical issues and were conducted in groups, e.g. discussing the comprehensibility of printed material. Having to write down their thoughts on cards or reading many e-mails in advance was experienced as exhausting.

From the researchers’ perspective, the patients were intensely involved in the current patient board. The only suggestion for improvement they could think of was giving the patients some additional written queries. The researchers did feel that patients should be involved in research decisions as they are the target group and as they indirectly funded the research via their taxes. At the same time, they were concerned that patients lacked the necessary methodological competences and wondered how this would work, and which competences patients would need for them to be able to assess research and participate in the necessary decisions.

Researchers described that they benefited more from actually attending meetings than from just reading the minutes of each meeting. Furthermore, having attended at least one meeting helped them to better understand the minutes of the meetings they had not attended. The minutes were described as valuable documents, which can also be used as a reference in the future. Patients did not discuss the value of having minutes.

#### Effort of PPI conduct

Patients reported that they did not feel burdened by their involvement. On the contrary, they would have liked to meet more often than two to three times a year. In contrast, the researchers experienced the PPI as very time-consuming. They however at the same time emphasized that the attendance was worth the effort (see category “benefits”). In addition to the actual attendance of meetings, researchers described the preparation and follow-up as “quite time-consuming, but it was fun” (R3:213).

#### Allowances

Patients acknowledged that the allowances were an incentive for participation, although some stated that they still would have participated even without any allowance. The amount of the allowance was rated as appropriate, “30 to 50 euros is what I find reasonable for a meeting [ …] with preparation” (P4:327). Researchers did not discuss patients’ allowances.

### Theme 4: benefits

Benefits of the involvement were described for the people involved, the trial itself as well as for future research.

#### Benefits to the people involved

Patients appreciated getting the opportunity to develop a deeper understanding of UTIs, and to learn about different projects, research results, and research in general. Researchers learnt about using language that is better understandable and less discriminatory for the patients. For example, patients preferred the term “to gain patients for a clinical trial” rather than “recruitment”, as, from their perspective, the latter seemed to objectify human beings. Participating in a patient board meeting was reported to have been an intense experience, “that was not a doctor-patient-relationship, but an entirely new situation, different from the ones I know” (R3:26). After their experience with the patient board, researchers reported that they were now “[ …] richer in experience and knowledge about what it, [PPI], can do to oneself and the trial” (R3:273).

#### Benefits to the research

While patients described benefits for themselves, they wondered about the actual impact of their input and the discussions in the patient board on research, “because for me it is not clear, what could be used at all” (P6:15). Some thought they were able to give stimuli for the researchers or generate ideas, e.g. for the recruitment of patients.

Researchers found that the PPI was of use for a range of aspects. They felt reassured that patients considered the aim of the research project to be of relevance. The patients made suggestions to the researchers regarding better ways to reach out to patients, and also gave them new ideas for the dissemination of results. Further, from the discussion of trial questionnaires, researchers learnt how question should be phrased for them to be easily understood while still being explicit. Researchers were also informed that patients would prefer questionnaires that also ask for positive changes, instead of focusing on negative experiences and symptoms only. Researchers summarized the benefits for the trial stating that “there were many details [ …] on which [ …] I certainly would not have come up with” (R3:19). In addition, researchers thought that the work with patients would be of use for the conception of future trials.

## Discussion

The aim of our study was to find out about the experiences and challenges of patients and researchers who were involved in a patient board for a RCT on UTIs in Germany. In the following, we will discuss our findings in the context of previous studies and highlight potential challenges we encountered. This is in accordance with Forsythe et al. and Morain, who demanded that researchers discuss the challenges they meet in the process of conducting PPI in order to improve its implementation in future ventures [14, 15]. The discussion is structured according to the four main themes that evolved during the study.

### Basis for cooperation

Patients and researchers found the atmosphere in the patient board to be pleasant. Mutual respect, appreciation of contributions, and cooperation at eye level were observed to be prerequisites for a good atmosphere. Although both patients and researchers were comfortable with the current composition of the patient board, their suggestions regarding groups of stakeholders that should be additionally involved to broaden the perspectives of the board differed. While patients suggested that women who had actually participated in the RCT on UTIs should also be involved, researchers suggested the involvement of members of the public who did not have experience with UTIs. Based on our preliminary research, we know that the possibility of being able to exchange with peers and scientists on their health issue motivates patients to get involved in PPI [12]. The suggestion made by researchers in the current study, that persons who do not share the experience of suffering from UTI be involved, might hence jeopardize this form of motivation.

Patients would have liked to learn more about their roles within the patient board at the beginning. They wished to know more about the aim of PPI in general, and of each meeting so they could better understand what was expected from them. This uncertainty might lead to the loss of important contributions, as patients may tend to express themselves according to the expectations they assume researchers or the moderator to have. However, patients should be able to influence the agenda as “researchers don’t know, what they don’t know” [7]. The issues that are brought up by patients and that researchers would not yet have considered can particularly challenge the assumptions of researchers and culminate in a learning process [29]. This insight resonates with the findings from other studies [29, 30]. Researchers employing PPI should thus be aware that explicit clarification of roles and aims is key to a fruitful involvement of the patients. In our study it was striking that this issue was discussed repeatedly by the patients during the focus group, whereas it did not come up even once during the focus group of the researchers.

Patients also suggested several ways to support the clarification of roles and aims. Besides being comprehensively informed at the initial contact and getting written information material, patients suggested that the issue of “roles and aims” be made an explicit topic in some or even every patient board meeting. We in fact did inform the patients at the initial contact about their roles and the aims of the patient board, gave them information material, and talked about how the PPI would be conducted at the beginning of the patient board. Their feedback however makes it clear that our approaches did not work well enough. One possibility could be that researchers, who also did not have extensive experience with PPI, were not able to give patients comprehensive information about roles and aims at this early time-point. While this could improve with increasing experience, other approaches might be more suitable. For instance, instead of using a one-directional information approach, a joint discussion could be used. This would enhance the clarification of roles and aims between patients and researchers and would also fit better to the idea of empowerment that PPI strives for. A joint discussion could hence lead to a common understanding of the aims of PPI as well as of the roles and responsibilities of all parties involved [11, 12, 31].

In our focus groups, patients were concerned that their involvement might not have an impact on the RCT. Other studies described researchers’ fears that PPI might jeopardize their projects, as it demanded a lot of time, and the ideas put forward by patients could not be implemented in the context of their trial [3]. It could therefore be helpful if patients and researchers also started talking about their uncertainties and concerns regarding their roles in PPI. These discussions might help reassure researchers as well as patients that not every issue mentioned by a patient needs to be incorporated into the study design. At the same time, suggestions that have an impact can be explicitly highlighted. While discussing this issue seems valuable to enhance mutual understanding and trust, it takes considerable courage to be able to talk openly about aims, hopes and fears [31].

*In summary*, we found that for a successful cooperation, enough time should be scheduled to discuss roles, aims, hopes and fears, during all phases of the project. A well-reflected composition of the people involved and a pleasant atmosphere are crucial to enable cooperation.

### Facilitation of PPI

Patients thought that getting acquainted and introducing own issues would be easier if they were involved from the start, whereas researchers thought that the introduction of own issues by the patients would develop over time with the help of the moderator. While it is understandable that patients need time to get acquainted before they feel confident to introduce their own issues, in the context of tightly planned trials, a long time of getting acquainted may lead to weak involvement. Therefore, stakeholders should reflect on how they can support patients to get familiar with the trial early, thereby empowering them to influence the agenda setting.

Similar to patients, researchers also needed time to get familiar with PPI. The experiences in the patient board in fact helped them to develop a more comprehensive understanding of what PPI is, and can be. Training for PPI might therefore be needed to support collaboration [31]. In the focus groups, the opinions regarding whether patients, researchers, or both should be trained for PPI, varied. On the one hand, training the researchers and not the patients would shift the responsibility almost completely from the patients and lead to reduced empowerment. On the other, extensive training sessions for patients would require more time, thereby possibly hindering the involvement of some patients.

While researchers thought of both groups’ training needs, patients only reflected on their own training needs. This may raise the question of whether patients felt that they needed to adapt to the researchers’ skills and way of doing research, instead of researchers adapting to the patients. Several studies demonstrated that both patients and researchers had training needs before conducting PPI. Researchers needed to learn how to facilitate PPI and how to work together with patients, while patients benefited from training through empowerment, gained more confidence and experienced an increase in knowledge and research skills [14, 30, 31]. As has already been mentioned, offering joint training sessions for researchers and patients could deepen their mutual understanding of working together. Finally, the need for training should be openly discussed at the beginning of any PPI.

Researchers found it difficult to always keep the patient board in mind for the duration of the RCT and the PPI did not become part of their routine procedures. This indicates that researchers might need to get more involved in the organization of PPI and regularly participate in and prepare meetings. Researchers could also be asked to send regular updates and questions to the patients in-between meetings to enable a deeper involvement for both sides.

*In summary*, to facilitate effective PPI, both patients and researchers need to be trained. Supporting patients in a way that enhances their confidence, knowledge and skills empowers them to actively shape the conduct and agenda of PPI. Researchers need to learn about PPI. Being involved in the organization of PPI might help them to incorporate PPI in their research routines.

### Organization and conduct

As has already been mentioned, both patients and researchers would have preferred the patient board to already start at the design stage of the trial. In our case, PPI was a research project with a defined timeframe, and the patient board was installed when the trial was already running. Implementing PPI only after the design stage of a trial may however be a common challenge, at least in Germany, as there is hardly any funding for PPI and no research culture of establishing PPI from the onset. How involving patients only after the design stage made it difficult for patients in our study to get familiar with their roles as well as how this led to researchers missing the opportunity to discuss essential decisions with patients has already been discussed in earlier sections. A possible solution for this dilemma, which has been tested as efficient in the UK, is the issuance of specific funding for the involvement of patients in the design stage of research projects [32, 33]. Stakeholders however also need to become more conscious about the relevance of PPI. Another approach would be the creation of a “generic” patient board that researchers could contact during the design stage to discuss their ideas with patients and members of the public respectively.

The opinions of patients and researchers differed regarding the optimal degree of involvement. Whereas patients wished to get more intensely involved and also preferred face-to-face-discussions over written queries, researchers could not imagine being more personally involved. This discrepancy is mirrored in the responses of the two groups on the issue of the level of effort associated with the involvement, which was perceived as being low by the patients, while researchers found the involvement to be more labor-intensive than they had expected. These findings underline the need to discuss roles, efforts, and ways of collaboration at the beginning of PPI.

In a similar study by Rhodes et al. [29], patients were observed to feel more visible, recognized, and empowered after they started to attend the steering group meetings of the project in addition to participating in the research advisory group. According to the authors, attending steering group meetings allowed the patients to directly experience the value of involving their perspectives in research. Patients in our patient board patients would also have liked to attend the meetings of the trial team in addition to the meetings of the patient board. They however clearly indicated that they would not expect all their recommendations to be followed and apparently felt that a more intense involvement on their side might lead to researchers being concerned about not having the final saying over the research project. Installing a shared mailing list between patients and researchers in which the patient board meetings are agreed, prepared, and followed up on, and where the intermediate status of the trial is regularly communicated and discussed might help to minimize some of the expected concerns and at the same time increase transparency.

*In summary*, it might be a common challenge for the organization and conduct of PPI that PPI is only installed after a trial has been approved for funding. Patients and researchers should discuss and agree on possible approaches in order to avoid unnecessary conflicts due to differences in perception of the intensity and effort of PPI.

### Benefits

Patients were interested to learn about the effects of their involvement, because making an impact was a factor of motivation for all of them [12]. Although a report on the present state of the trial and how the results from the last patient board meeting were used was given at the beginning of each patient board meeting, when asked about their impact on the trial in the final focus group, patients stated that it was not clear to them if their involvement had made any difference to the trial. However, at the same time they described how they had personally benefited from the involvement. This apparent discrepancy is probably be due to the fact that during the initial phase of our study, researchers could not yet identify, less alone articulate, the (potential) benefits of the patient board. Their awareness on this gradually developed with their participation in the focus group discussion.

The importance of communicating the actual impact of PPI has also been identified in other studies. For instance, Doria et al. state that the benefit of PPI should be “clearly identifiable both to the research team and to the patient contributors” [34]. This, as shown by our findings, might however be easier said than done. Nonetheless, it remains a fact that researchers can only inform patients about the impact of their involvement if they themselves are aware of the benefits. It is also important that all participants, be they patients or researchers, be made aware that PPI does not necessary lead to striking modifications, but often operates through more subtle changes such as an increased awareness for the perspective of patients. Further, Staley found that the knowledge and skills gained by researchers through PPI later changed how they conducted research [7].

*In summary*, making sure that all parties involved are aware of the benefits of PPI is essential to maintain their motivation. This might be challenging if all individuals involved are unfamiliar with PPI and still need to develop their awareness on the impact of PPI.

### Strengths and limitations of this study

We found the patient board to be a good method to bring patients and researchers together and discuss the conduct of the UTI trial. Patients and researchers involved in the patient board perceived this experience as fruitful and productive.

As it is not easy to share the information that one is suffering from a UTI with strangers, we only involved women a history of UTI as ‘patients’ in our patient board, who openly discussed their experiences. Only women were included in the PPI because the underlying RCT also included women only. We do not know if the discussions would have been as open had the group comprised men and women. Further, we do not know if the outcome would have been different had we included women who had never suffered from UTI.

While conducting the focus groups separately for patients and researchers served to avoid mutual influences, a joint focus group might have offered a platform where patients and researchers could have compared their experiences. However, as the team of authors comprised members from both groups, experiences from both could be fully incorporated in the manuscript.

Only three researchers participated in the focus group on experiences, and two of them had attended only one meeting of the patient board. Therefore, our findings about researchers’ experiences come from a small number of people, most of whom had limited experience with the board, and the findings may overrepresent the experience of the one researcher who attended all board meetings. This may affect the generalizability of the results.

The fact that some of the authors had double functions could be perceived as a limitation. For instance, IS not only moderated the meetings of the patient board but was also involved in the data analysis. While this arrangement on the one hand enabled a deeper understanding of the patient board, it might on the other hand have affected the impartiality of analysis.

Lastly, we reported the benefits that patients and researchers perceived from getting involved in the patient board but did not assess the actual impact of PPI on the conduct of the RCT as this was beyond the scope of the study.

## Conclusions

- Involving patients in research is increasingly recognized as a prerequisite for conducting meaningful, high-quality research.
- To facilitate the conduct of effective PPI, resources in terms of staff, time and finances, adequate working and communication structures, and training for patients and researchers are needed.
- Early involvement of a patient board in research projects will allow for closer collaboration and continuous exchange of ideas.
- Especially at the beginning of PPI, care should be taken that patients and researchers agree on their roles and the mode of cooperation.
- The expectations, motivations, perceptions, and experiences might differ between patients and researchers; this should be reflected during the conduct of PPI.

## Supplementary information

**Additional file 1.** COREQ (COnsolidated criteria for REporting Qualitative research) checklist

**Additional file 2.** GRIPP2 checklist

## Data Availability

The datasets generated and analysed during the current study are not publicly available due to data protection regulations. This is part of the guarantee the patients and researchers received before they gave informed consent.

## Abbreviations

COREQ: Consolidated criteria for reporting qualitative research
PPI: Patient and public involvement
RCT: Randomized controlled trial
UTI: Urinary tract infection
